# What primary care physicians in Germany consider meaningful and meaningless work: a qualitative interview study using reflexive thematic analysis

**DOI:** 10.1186/s12875-026-03495-z

**Published:** 2026-08-01

**Authors:** Joséphine-Charlotte Coleman, Alina Stortz, Matthias Stadler, Martin R. Fischer, Marion Huber, Katharina Schütte-Nütgen, Tago L. Mharapara, James R. Greenslade-Yeats, Matthias J. Witti

**Affiliations:** 1 Institute of Medical Education, LMU University Hospital, LMU Munich, Pettenkoferstraße 8a, Munich, 80336 Germany; 2 School of Health Sciences, ZHAW Zurich Universities of Applied Sciences, Zurich, Switzerland; 3 Department of Acute and Emergency Medicine, Elisabeth-Hospital Essen, Essen, Germany; 4 Faculty of Medicine, University of Münster, Münster, Germany; 5 Faculty of Business, Economics and Law, Auckland University of Technology, Auckland, New Zealand

**Keywords:** Primary care physicians, General practitioners, Pediatricians, Meaningful work, Meaningless work, Job satisfaction, Reflexive thematic analysis

## Abstract

**Background:**

Germany faces a critical shortage of primary care physicians (PCPs). A protective factor against job turnover is the experience of meaningful work. While the presence of meaning fosters work engagement and well-being, its absence can lead to career exit. This qualitative study explores perceptions of meaningful and meaningless work among German PCPs to inform health policies and help minimize career exit.

**Methods:**

We implemented a qualitative study design, conducting online, one-on-one, semi-structured interviews with 24 PCPs practicing across Germany. Participants were recruited through purposive and snowball sampling to ensure a representative sample. We evaluated the data using reflexive thematic analysis.

**Results:**

We generated five central themes. Meaning is derived from realizing professional identity through autonomous medical expertise (theme 1) and cultivating trust-based, long-term patient relationships on an equal footing (theme 2). Conversely, meaninglessness arises through unrealistic patient expectations and physicians’ experiences of frustration (theme 3), and systemic devaluation through administrative burdens and inadequate compensation (theme 4). The resulting disillusionment (theme 5) increases the risk of career exit.

**Conclusions:**

Our findings show that the projected PCP shortage in Germany may stem in part from a “crisis of meaning”. Current billing structures prioritize high-volume transactional encounters, which obstruct the very tasks that PCPs find meaningful. To ensure workforce stability, policy reforms must move beyond individual resilience and address these systemic drivers of meaninglessness.

**Supplementary Information:**

The online version contains supplementary material available at 10.1186/s12875-026-03495-z.

## Background

Primary care physicians (PCPs) play a fundamental role in healthcare systems worldwide, frequently serving as “gatekeepers” for specialist referrals [1]. We define PCPs in the German context as general practitioners (GPs) and pediatricians in private practices (PPs) [2]. In contrast to physicians in other outpatient practices and specialties, PCPs in Germany are typically the first point of contact for a broad range of concerns, extending beyond acute medical care. They oversee the coordination and management of diagnostics and treatment and provide continuous, comprehensive care [1, 3].

Nevertheless, it is important to acknowledge that Germany’s primary care system is currently only moderately developed compared to international standards [4]. In particular, a European comparison of ten primary care dimensions shows that Germany ranked 30th of 31 countries on care coordination, which includes the gatekeeping function and teamwork of PCPs [4]. Patients in Germany enjoy an unusually high degree of autonomy in their choice of physician by international standards within a healthcare system that is otherwise complex and fragmented, comprising more than 90 statutory health insurance funds alongside a separate private insurance system [1, 5]. This indicates a need for considerably stronger political and structural support for care coordination to be fully realized [4].

These structural characteristics may contribute to increased administrative burden, perceived system inefficiencies, and therefore physician dissatisfaction and job turnover [6]. These factors have plausibly contributed to a noted decline of GPs in Germany, with a projected shortage of approximately 11,000 GPs by the year 2035 [3, 6]. One explanation for this could be the strenuous working conditions experienced by GPs in Germany. They have an average weekly caseload of 253 patients, work approximately 53 h, and spend about 8 min per consultation [7]. In other words, the number of patients seen by German GPs is twice as high as that of their counterparts in other Western countries, such as Sweden, Canada, and the United Kingdom [7]. Further reasons for job turnover could be a lack of autonomy and a sense of being undervalued, particularly as a result of the complex remuneration structures imposed by the German billing system [8, 9].

An important protective factor for career commitment that also counters work absenteeism and turnover intention is the experience of meaning in one’s work [10, 11]. The concept of meaningful work can be defined by three dimensions: significance (the intrinsic value one assigns to one’s work), comprehensive purpose (the feeling of being part of a larger whole that goes beyond purely economic goals), and self-actualization (the opportunity to contribute and develop personal potential) [12]. As will become apparent in our findings, physicians’ accounts of meaningful work were frequently articulated in terms of their sense of their role as professionals. We therefore draw on the concept of professional identity to aid interpretation, defined as “a representation of self, achieved in stages over time during which the characteristics, values, and norms of the medical profession are internalized, resulting in an individual thinking, acting, and feeling like a physician” [13].

The presence of meaningful work is positively associated with work engagement and commitment, job satisfaction, and mental health [11, 14–16]. Physicians already experience mental health problems such as burnout and suicidality, which can have detrimental effects on the quality of patient care, as evidenced by an increase in medical errors, and a decrease in patient safety and satisfaction [17–19]. Furthermore, it affects health care systems, leading to reduced productivity and increased turnover [19].

Considering this evidence, an important question is: Which aspects of everyday work life are perceived as particularly meaningful or meaningless by PCPs? To date, research on physicians’ perception of meaningful work has been conducted through international studies in Norway, Japan, and Saudi Arabia [20–22]. However, to our knowledge, no qualitative interview studies on the topic of meaningful and meaningless work have been conducted with German PCPs. Therefore, the goal of this study was to clarify PCPs’ perceptions of meaningful and meaningless work in Germany, to inform future health policies aimed at reducing the rate of career/role attrition.

## Methods

### Study design

We implemented a qualitative study design to gain deeper insights into PCPs’ perceptions of meaningful and meaningless work. We conducted online, semi-structured interviews, and employed reflexive thematic analysis (RTA) to evaluate the data [23].

This study is part of a multicenter, cross-national project on meaningful and meaningless work in cooperation with researchers at the Auckland University of Technology in New Zealand and Karolinska Institutet in Sweden, in which a shared interview guide was used across countries to ensure comparability of data collection and analysis.

### Sampling and recruitment

In Germany, PCPs include general practitioners (GPs), either trained in family or internal medicine, and pediatricians working in private practices (PPs) [2].

We purposively sampled PCPs practicing in Germany to obtain a representative sample of the population while ensuring a balanced distribution of gender and region. Authors Joséphine-Charlotte Coleman (JCC) and Alina Stortz (AS) contacted organizations that could potentially support recruitment (e.g., institutes of general medicine, medical associations, pediatric and general practice societies) nationwide via email. These emails included a summary of the study and an invitation to participate. The correspondents were requested to disseminate this information through their network of PCPs. Once this pathway was exhausted, JCC and AS contacted PCPs directly via email, and a snowball sampling method was used to reach additional PCPs. Most PCPs contacted did not respond to the emails, and only a few actively declined the invitation, with many citing time constraints as the reason for their non-participation. Once an interview with a PCP had been arranged, they received a personal letter outlining the study’s details and a consent form allowing us to interview and record them and to use the obtained data for analysis. They were required to sign and return the form prior to the interviews.

### Data collection

JCC and AS conducted semi-structured interviews between April and October 2025. The interview guide employed in this study was adapted from a guide developed by researchers in New Zealand. The original guide was in English and was subsequently translated into German using the TRAPD (translation, review, adjudication, pretest, and documentation) method [24]. The interview guide consisted of open-ended questions addressing a broad range of topics, including physicians’ background and everyday work, meaningful and meaningless work, coping strategies for meaningless work, and the perceived influence of the German healthcare system on the experience of meaning. As this study’s research questions concern physicians’ perceptions of meaningful and meaningless work specifically, we restrict our reporting here to the three corresponding topic blocks: (1) backgrounds and experiences, (2) meaningful work, and (3) meaningless work. A summary of these three topics is provided in Table 1. The coping and healthcare-system question blocks were analyzed separately and will be reported in a companion publication. The translated German interview guide was piloted with a general practitioner and a pediatrician. JCC and AS conducted the interviews in German and audio-recorded them via the Zoom software platform independently of one another. Subsequently, the interviews were transcribed verbatim, and the data were anonymized [25, 26]. During each interview, only one researcher (either JCC or AS) and the participant were present. In total, 24 PCPs participated in the study. No repeat interviews were conducted. Participants were offered the opportunity to review their interview transcript prior to analysis. Four participants made use of this option, and no substantive changes to the transcript content resulted.

**Table 1:** Summary of interview guide topics relevant to the present study

1. Introduction	
1.1 Career	Could you please give me an overview of you career? E.g., career span, specialization, further qualifications
1.2 Everyday work and practice structure	Could you please tell me about your current work as a GP/pediatrician? E.g., typical working week, workload, frequent medical consultations and treatments, patient population, practice setup and structure.
2. Meaningful work	
2.1 Perception of meaningful work	Thinking about the las week or month of work, please tell me about one or more situations in which you experienced work as meaningful? Why did these situations feel meaningful? What were the outcomes of meaningful work on yourself, your family, your patients, and your practice?
3. Meaningless work	
3.1 Perception of meaningless work	Thinking about the last week or month of work, please tell me about one or more situations in which you experienced work as meaningless? Why did these situations feel meaningless? Who suffers when work feels meaningless - e.g., you, your family, your patients, and/or your practice?

### Data analysis

We conducted a reflexive thematic analysis (RTA) to analyze the data [23].This allowed us to systematically examine not only the concept of meaningful and meaningless work, but also how it is shaped by and implemented in the context of everyday working life.

We applied the established six-step RTA approach. Starting with familiarization, JCC and AS initially transcribed and annotated their own interviews and subsequently read and annotated the other researcher’s interviews. Following this, JCC and AS coded all the interviews together and generated themes from the codes. Collaborative coding of the interviews deepened reflexive analysis by requiring the coders to articulate and reconcile their diverse perspectives. Additionally, we held reflective sessions within the research team (JCC, AS, MS, MF, MH, KSN, and MJW). We established a list of codes, themes, and their corresponding definitions, which we continuously revised throughout the analytical process. The exemplary quotes were translated into English. The software MAXQDA 24 was used for data analysis [27]. We used the Reflexive Thematic Analysis Reporting Guidelines (RTARG) developed by Braun and Clarke for reporting our data [28].

### Reflexivity

When using reflexive approaches to analysis, researchers must be aware of their central role in interpreting their data [29]. JCC and AS both have backgrounds in medicine and experience in primary care practices, gained through internships and lectures. They repeatedly examined and reflected on their own assumptions with the aim of understanding their influence on the analytical process and the results. This involved questioning whether their own frustrations with the German primary care system influenced the interpretation.

### Ethics

This study was approved by the ethics board of the medical faculty of the Ludwigs-Maximilians-University Munich and was performed in accordance with the ethical principles of the Declaration of Helsinki (World Medical Association).

## Results

We interviewed a total of 24 participants. The complete set of participant characteristics is presented in Table 2. The interviews lasted between 20 and 60 min. We generated five themes, 34 codes, and 332 segments. The themes and codes are shown in Table 3.

**Table 2:** Study population description

Gender	Female	13
	Male	11
Specialty	General Practitioner	17
	Pediatrician	7
Type of Practice	Solo Practice	6
	Group Practice	15
	Ambulatory Healthcare Center	3
Years of Experience	< 10 years	2
	10–20 years	7
	20–30 years	8
	> 30 years	3
	Not mentioned	4
Years of Experience in private practices	< 10 years	10
	10–20 years	6
	> 20 years	6
	Not mentioned	2
Region	Northern Germany	3
	Southern Germany	11
	Western Germany	6
	Eastern Germany	4
Town Size	Village	3
	Small Town	7
	Large Town	6
	City	8

**Table 3:** Themes and codes

Theme	Codes
Realizing professional identity through applying medical expertise	Medical thinking
	Adequate treatment of acute and chronic illnesses
	Weighing benefits and risks
	Practicing preventative care
	Visible therapy results
Meaning is found in relationships and trust	Contact with patients in general
	Doctor-patient-relationship based on equality
	Alleviate patients’ concerns and fears
	Support patients beyond medical issues
	Establishing and holding the patient’s trust
	Long-term care relationships
	Receiving patient gratitude
	Patients are satisfied with treatment
	Feedback increases meaning
	Cooperative teamwork within the practice
	Task delegation
Meaning may be diminished when unrealistic patient expectations and physicians’ experiences of frustration overshadow the medical consultation	Negatively perceived patient encounters
	High patient expectations
	Instrumentalization by patients
	Insufficient health literacy among the general population
	High input, low outcome
Loss of professional autonomy and devaluation of medical expertise	Cooperation with health insurance companies
	Healthcare system’s economic constraints
	Limited autonomy
	Health insurance companies are detached from reality
	Required documentation due to institutional mistrust
	Inadequate financial compensation
	Bureaucratic duties
	Non-medical duties
Disillusionment is a risk factor for leaving the profession	Reality differs from perception of profession
	One’s own quality standards cannot be fulfilled
	Thoughts of leaving the profession

### Theme 1: Realizing professional identity through applying medical expertise

The participating PCPs experience meaningful work by applying their medical expertise and skills. This includes autonomous medical problem-solving, developing differential diagnoses, and making therapy decisions based on those diagnoses. It is not only viewed as a professional task, but as an expression of professional identity. Meaning arises when medical knowledge is applied autonomously, reflected upon responsibly, and experienced as effective.

The participants understand this to be the core of medicine. They associate it with the knowledge and skills they acquired during their undergraduate medical studies. Medical thinking, understood as a problem-based, analytical approach that takes individual patient situations into account, forms the basis of professional identity.

*Yes*,* so my work always feels meaningful when I have individual patients who have a problem or an issue that is also interesting to me*,* where I can talk about it calmly*,* where I perhaps also need to take a closer look*,* what can I figure out*,* what can be behind it […] GP13*.

The adequate treatment of acute and chronic illnesses, as well as the reliable recognition of emergencies, are ways to express medical competence and are perceived as meaningful by the participants.

*[…] also*,* of course*,* when one can accurately assess acute situations*,* refer patients in emergency situations accordingly*,* or provide further inpatient or outpatient care […] GP16*.

Furthermore, the weighing of benefits and risks was consistently mentioned during the interviews. For example, in the context of medication and decision-making regarding hospitalization, meaningfulness derives from the guiding principle “do no harm” and from critically questioning medical procedures.

*Then I find averting harm from patients highly meaningful. This means questioning ongoing medication. GP8*

*What is also highly meaningful is (…) regarding hospitalization*,* a good assessment*,* how much harm and how much benefit do I get from hospitalization? It’s worth taking the time to call the hospital in advance and such things. GP8*.

Not only curative care, but also preventative care play a role in attributing meaning to medical practice. Participants describe discussions about health care, prevention, and lifestyle as personally significant, even if their effects are often limited or only visible in the long run.

*[…] Discussions about health care*,* prevention*,* nutrition*,* things like that. I enjoy talking with patients about these things. (…) If I advise 100 patients*,* maybe two will come back at some point [and] will implement them. That doesn’t stress me out*,* I find it […] I would still continue to do so because I think that*,* if two implement it*,* that’s still very good. That would make me happy. GP16*.

Visible therapy results reinforce the feeling of medical self-efficacy. These experiences of success strengthen one’s confidence in one’s medical skills and consolidate one’s professional identity. Therefore, it increases the feeling of meaningfulness.

*And if the health markers improve significantly*,* that’s something that makes me happy*,* because I can see that my work is somehow successful. GP16*.

The results show that meaningfulness arises primarily when analytical and reflective decision-making is applied, and treatment outcomes are visible. This helps to form professional identity. At the same time, both curative and preventive approaches contribute to meaningfulness, provided they are perceived as expressions of professionally sound action. This forms the basis for a solid doctor-patient relationship, which is described as an independent source of meaningfulness in the second theme.

### Theme 2: Meaning is found in relationships and trust

A trusting doctor-patient relationship based on equality, encompassing both professional and emotional components, was described by participating PCPs as a central aspect of meaningfulness.

*I find my work most meaningful when I have contact with patients and when there is a relationship. That’s really great. GP13*.

A balanced relationship between PCPs’ medical commitment and patient cooperation is identified as a prerequisite for a functioning relationship. Participants find it particularly fulfilling to alleviate patients’ concerns and fears and to support them even beyond medical issues.

*So, as a GP, you have many, many positive situations at least, because you can always […] do something meaningful, help patients, alleviate their worries, alleviate their fears. GP4.*

*If, for example, parents with a young child are uncertain about how to deal with nutrition or common infections or such, then it’s meaningful. PP7.*

*So, where I have experienced a lot of gratitude, for example, when you get involved, that is, when you stand up for the patient. GP15.*

Long-term care relationships enable continuity, trust, and a deeper understanding of individual circumstances, further increasing the meaningfulness of patient contacts.

*Well, basically it’s a feeling of resonance, meaning that you are on the same level as the patients and feel comfortable with them, and ultimately feel that you are working on the essential [issue]. GP9.*

*But that’s the beauty of general practice, it’s this relationship, this long-term relationship that you enter into. GP9.*

Simultaneously, recognition and appreciation of medical expertise, as reflected in patient feedback and gratitude, contribute to the emotional confirmation of one’s professional identity. These aspects promote meaningfulness and balance in a mutually beneficial partnership.

*It is meaningful and fulfilling when parents feel well advised and [respond accordingly]. PP6.*

*Then I sometimes have very elderly people*,* perhaps even at the end of their lives*,* who are always incredibly grateful if you take care of them*,* and come and write down the things they need. I make an effort to ensure that they are still comfortable at home for as long as possible*,* or even in the final stages. I find that meaningful. GP13*.

Not only relationships between physicians and patients, but also within the practice team, contribute to meaning at work, according to the PCPs interviewed. Working within a cooperative team, as well as the possibility of delegating tasks to colleagues, add to the distribution of responsibility, thereby reducing the burden on the individual.

*It’s a relatively tight schedule, but the team in (city) is very experienced. They do a great job preparing everything so that I can really focus on the medical side of things. PP2.*

*So, I always try to engage in open dialogue for the benefit of our practice and the team […] to create a harmonious working environment. And when the employees are satisfied overall, that makes me happy too. Or rather, it makes me satisfied and gives me a sense of meaning. GP16. *

The participants’ statements show that meaning arises when the physician’s role is not reduced to functional care but is integrated into a trusting, appreciative physician-patient relationship on an equal footing. Meaningfulness is further strengthened by cooperation and shared responsibility within the team.

### Theme 3: Meaning may be diminished when unrealistic patient expectations and physicians’ experiences of frustration overshadow the medical consultation

Participating PCPs reported a loss of meaning when the physician–patient partnership no longer reflected an equal and balanced relationship. For example, some patients’ expectations are perceived by participating PCPs as high or inappropriate. This is exemplified by patients who seek medical care or diagnostic procedures that are not clinically warranted, or who expect immediate access and responsiveness from their PCP despite organizational and personnel constraints that make such responsiveness unattainable. This can lead to a shift in the PCPs role toward a more service-oriented function, with physicians reporting a sense of instrumentalization.

*I think that many of us experience this, that many, including younger patients, many patients in general, have high expectations, and of course these can increasingly no longer be satisfied (…) and it doesn’t make sense to satisfy them. GP17.*

*I always feel that work is meaningless when I feel I’m being taken advantage of and exploited to obtain an unjustified sick note or long-term sick-leave or retirement or something like that. […] When I feel like I’m being instrumentalized. GP3. *

However, it is important to note that these expectations may in fact be meaningful and important requests for the patients themselves.

*Because the physician’s perception of what is meaningful and the patient’s perception of what is meaningful*,* those two are far apart. GP8*.

Additionally, insufficient health literacy among the general population is described as burdensome. Frequent consultations for trivial symptoms, as well as identical or recurring requests for advice, contribute to an increased workload and are perceived as inefficient.

*[…] because people are increasingly somatizing and are mentally ill, they experience even mild symptoms as life-threatening and no longer have any health literacy and cannot really differentiate and assess them properly. GP16.*

*In some cases, it’s also this feeling that we are putting in a lot of effort (.) for a less than satisfactory outcome. […] GP17. *

Situations in which physicians feel instrumentalized are particularly stressful. Patients’ high expectations can limit physicians’ self-efficacy and promote alienation from their professional identity. The discrepancy between the time and energy invested and the perceived impact reinforces the feeling of loss of meaning.

### Theme 4: Loss of professional autonomy and devaluation of medical expertise

In the interviews, cooperation with health insurance companies and economic constraints of the healthcare system are described as significant limitations to the physician’s autonomy. Decision-making processes seem to be controlled by external factors. This leads to a sense of frustration, a lack of appreciation, and reduced self-efficacy among participants.

*And I believe it doesn’t matter who you talk to. This is what drives people crazy. That it’s not about the patient at all. At no point are the health insurance companies concerned about the patient. NEVER. PP1*.

One such example is the requirement made by some employers to submit a sick note from the first day of absence. Patients suffering from, for example, a minor cold are still required to present themselves, despite the fact that such a visit is not medically necessary from the perspective of either the physician or the patient.

*So*,* what I consider meaningless is when patients come in with a cold needing a sick note from us. I find it rather pointless that I need to see them just for a sick note. GP1*.

The participants perceive the need to justify medically necessary decisions to health insurance companies as a form of detachment from reality on the companies’ part.

*Health insurance inquiries. When you have a patient in her early 30s with metastasized breast cancer and you have to argue with the insurance company about why you’re putting her on sick leave for chemotherapy. So*,* it’s just something where I think*,* listen*,* you’re desk workers*,* you have no idea about real life and you’re telling me how to handle things. […] GP10*.

Not only is the detachment from reality criticized, but also the high level of documentation required, which participants feel is unnecessary. Non-medical activities, especially administrative and organizational tasks, tie up considerable resources and are described in the interviews as a key aspect of meaningless work.

*And now I’ve recently been spending half the morning on weekends*,* on Sunday*,* doing that. The meaningless thing is that I have to pseudonymize all the patient names*,* I have to code everything*,* I sit there and delete the names from all the documents and then write in the code they want for them and so on*,* and it’s meaningless. So that’s my time wasted. GP4*.

Additionally, some even perceive it as an expression of institutional mistrust directed towards physicians.

*Yes*,* on those afternoons when I sit there after office hours (laughs) and somehow always have a documentation workload for numbers*,* which are only worth a few euros*,* just in case I’m audited. To justify it*,* basically. So*,* this fundamental mistrust of doctors on the part of health insurance companies. GP13*.

Furthermore, poorly or unpaid medical services are perceived as devaluing physicians’ work. Time-consuming diagnostics or consultations are not adequately compensated financially, which only reinforces the feeling of structural injustice.

*Or for example*,* you offer something*,* which only few people can do*,* such as abdominal ultrasounds. We don’t just do heart [ultrasounds]*,* but the rest of the child as well. It’s just so poorly compensated that you don’t do it anymore. I can’t invest half an hour for 13 €. I can’t do that. PP4*.

This leads to a discrepancy between the individual’s ideal image of a physician and the reality of medical practice.

### Theme 5: Disillusionment is a risk factor for leaving the profession

The ideal-reality discrepancy mentioned in Theme 4 against the backdrop of Theme 1, as well as the discrepancies between Theme 2 and 3, lead to disillusionment among some participating PCPs.

*This means my wish is, or I became a doctor, because I, as simple as it sounds, I wanted to help people. Yes, that was my biggest “why”. […] Unfortunately, in everyday medical practice, that often goes out the window because I am often a service provider and / or I also issue sick notes for people where perhaps other interventions might be possible. GP16.*

*The entire billing process is meaningless. It is degrading for someone who has studied for years and trained as a specialist to have to consider whether the meningococcal B vaccination is the first, second, or third and whether there is an AZ or B at the end of the number. That is degrading. GP7. *

Original motives, such as the desire to help people, are increasingly overshadowed by administrative requirements and systemic constraints that are time-consuming yet not perceived as meaningful. This prevents quality standards from being met, leading to a sense of moral and cognitive dissonance.

*Yes*,* that’s a really sad reflection on the situation. Because you simply don’t have the chance to deliver the quality that you yourself would like to deliver. GP8*.

This persistent discrepancy leads to a loss of professional identity and can encourage thoughts of leaving the profession.

*But I try not to dwell on it too much anymore, because I can’t change it now, but it will slowly lead to me not doing this job for the rest of my life. GP16.*

*And that’s why you should never become a doctor, even if it’s the best job in the world. But everything else about it is SO miserable*. *PP1. *

Loss of meaning appears not as a momentary burden, but as a structural risk for long-term withdrawal from the profession.

## Discussion

This is the first qualitative study to identify the specific drivers of meaningful and meaningless work among German PCPs. We generated two themes that describe the perception of meaningful work: realizing professional identity through applying medical expertise, and finding meaning in relationships and trust. Meaningless work was expressed through the following systemic themes: meaning may be diminished through unrealistic patient expectations and physicians’ experiences with frustration, and loss of professional autonomy and devaluation of medical expertise. Theme 5 “Disillusionment as a risk factor for leaving the profession” is a consequence of the greater impact of meaningless situations and tasks than meaningful ones.

The study’s findings align with and expand the limited existing international research regarding the perception of meaningful work among PCPs. Similar to findings in Norway, our participants identified meaningful work through applying medical expertise [20]. This finding can be situated within the concept of professional identity, most commonly discussed in relation to its formation during training [13]. Our data suggest that for established PCPs, meaning depends less on forming this professional identity than on the ongoing ability to enact it in daily practice. This includes applying expertise autonomously, exercising clinical judgment, and seeing the results of one’s own decisions. However, there is a divergence when considering the international structural contexts of the findings. German GPs operate within a system of higher patient volume, longer working hours, and shorter consultations times compared to other countries, such as Sweden, Canada, and the United Kingdom [7]. Therefore, activities identified as meaningful and professional identity sustaining are becoming impossible to perform, creating a volume versus meaning tradeoff, where high patient load kills meaningful work.

While high workload can be a factor influencing career exit, our findings indicate that the nature of the workload is important as well [8, 9]. PCPs derive meaning from preserving their professional identity and cultivating trusting, balanced relationships. However, this is limited by a loss of autonomy and control, as well as devaluation of medical expertise due to institutional and systemic prerequisites, factors that also encourage career exit [8, 9]. According to our data, the current primary care reimbursement structure in Germany, which is characterized by high-volume, low-duration encounters, directly obstructs the two primary buffers against meaninglessness: the application of medical expertise and the cultivation of trust-based patient relationships. It further devalues the profession, burdening it with illegitimate tasks, and limits professional autonomy, thereby contributing to a loss of meaning and possibly encouraging job turnover [8, 9]. A fee-for-service system, such as used in Germany, where physicians are responsible for their own salaries and for keeping their practices financially afloat, might hinder the implementation of meaningful tasks. Policy interventions must do more than reduce bureaucracy; they must re-legitimize the diagnostic complexity and relationship-building that PCPs find meaningful. Reforming the German Uniform Assessment Standard (Einheitlicher Bewertungsmaßstab, EBM) in favor of relationship-based billing would effectively institutionalize the trust-based patient relationships identified in our themes as a primary buffer against burnout. By shifting from a volume-based to a quality- and relationship-based payment model, PCPs would have the temporal flexibility and financial autonomy necessary to engage in the psychosocial and diagnostic complexity they find meaningful, and continue realizing the professional identity that drew them to the profession in the first place.

Furthermore, while PCPs value trust-based relationships with patients, they struggle with what they perceive as frustrating patient encounters. One solution would be to shift the burden of providing patient education on simple health and lifestyle topics away from PCPs and onto policymakers, investing more in preventive healthcare, and making healthcare education programs available to the general population without any obstacles.

Experiencing meaning in one’s work is an important protective factor against job turnover [9, 10]. The preservation of physicians’ mental health is not merely an occupational health issue but a fundamental prerequisite for a resilient healthcare system. A loss of meaning can have negative effects on mental health, which in turn implicates reduced patient safety and increases healthcare costs [17–19]. Further implications for patient safety arise from the data’s revelation that meaninglessness is precipitated when structural constraints prevent PCPs from upholding their professional quality standards. This systemic loss of meaning ultimately culminates in disillusionment, which our data identify as a driver of career-exit thoughts among PCPs. Therefore, our findings suggest that the projected shortage of 11,000 GPs will not only be driven by retirement but by an increasing loss of meaningful work [4]. If healthcare systems cannot protect the activities PCPs find meaningful, the shortage crisis will accelerate. Health policies must therefore move beyond individual resilience and tackle the issues at a systemic level [30].

A primary strength of this study lies in its qualitative design, which enabled a nuanced exploration of the constructs of meaningful and meaningless work that quantitative tools often miss. Furthermore, we achieved a diverse and representative sample across gender, regions, ensuring that the findings reflect a broad spectrum of the German primary care landscape.

However, several limitations are to be considered. First, the study was conducted within the German healthcare system. The findings are therefore context-specific and may not be directly transferable to other healthcare systems. Nevertheless, while German PCPs are particularly affected by high workloads and limited patient time, these challenges are shared by PCPs worldwide [7]. The alignment of our results with broader European trends suggests that these findings may have relevance beyond the Germany healthcare system and provides a trajectory for future international comparative research.

Second, the study used purposive and snowball sampling. This may result in self-selection bias, making it likely that the most severely strained physicians were unable to participate, which could make our findings a conservative estimate of the current crisis. Conversely, the snowball method could lead to a homophile bias, where participants recommend colleagues with similar frustrations. However, given that we needed to access PCPs with something useful to share, our sampling method was a necessary pragmatic step to reach as many potential participants as possible.

Third, we did not explore whether PCPs who are practice owners had a different understanding of meaningful and meaningless work than employed PCPs, as the former might have a higher administrative burden and might therefore be more affected by a loss of meaning. However, our findings demonstrate the meaninglessness goes beyond administrative tasks, affecting all PCPs.

Fourth, while this paper briefly mentions a divergence of perspectives between PCPs and patients regarding adequate expectations, it does not explore this divergence in depth. This is because the paper’s focus is on the perspective of PCPs, and delving deeper into the topic would exceed the paper’s scope. Consequently, we recommend that further exploration of this subject from the patients’ perspective is a suitable avenue for future research.

## Conclusion

This study suggests that the increasing shortage of PCPs in Germany cannot be attributed entirely to high workload or a lack of autonomy and recognition. A lack of meaningful work and a rise in meaningless tasks also contribute. While PCPs find meaning in applying medical expertise and cultivating patient relationships, the current German healthcare structure hinders this. The current fee-for-service model, which prioritizes high-volume, transactional encounters, devalues these meaningful tasks and experiences, and imposes a considerable burden on PCPs.

Our findings suggest that unless health policy moves beyond building individual resilience and preventing mental health issues, the job turnover will further increase. The need to reform the German Uniform Assessment Standard (Einheitlicher Bewertungsmaßstab, EBM) to incentivize psychosocial complexity and diagnostic depth is paramount to ensure the resilience of the healthcare system. Addressing these challenges through systemic policy recalibration rather than individualized psychological support is essential for long-term workforce stability.

## Supplementary Information

Supplementary Material 1: Interview guide

## Data Availability

The datasets generated and analyzed during the current study are not publicly available due to restrictions imposed by the ethics approval governing this study, as the interview transcripts contain sensitive qualitative data that cannot be shared openly, but are available from the corresponding author on reasonable request.
