# Supplementary material for: What primary care physicians in Germany consider meaningful and meaningless work: a qualitative interview study using reflexive thematic analysis
Source: BMC Prim Care. 2026 Aug 1;27:287. doi: 10.1186/s12875-026-03495-z (PMC13429019; doi:10.1186/s12875-026-03495-z)
Supplement: Supplementary file 1 — Supplementary Material 1: Interview guide [file 12875_2026_3495_MOESM1_ESM.docx]

**Interview Guide: What do primary care physicians in Germany consider meaningful and meaningless work? A qualitative interview study using reflexive thematic analysis.**

The following table shows the interview guide used, including key questions (in bold) with follow-up questions.

| Outline | Key questions with follow-up questions |
| --- | --- |
| 1. General information |  |
| 1.1 Career overview | - **Could you please give me an overview of your career as a GP/pediatrician?** - How long have you been in this profession? - Which medical specialization do you have and/or what specialist training(s) do you have? |
| 1.2 Everyday work and practice | - **Please tell me about your current work as a GP/pediatrician. Please describe a typical working week as a GP/pediatrician.** - Workload (How many patients do you see per day/week?) - Types of medical consultations and procedures that are most commonly attended to - Patient base - Practice setup (e.g., colleagues, individual or group practice) - If you work in a group practice, what is your role (e.g., head of the practice or employee) in the practice? |
| 2. Meaningful work | |
| 2.1 Meaningful work | - **Thinking about the last week or month of work, please tell me about one or more situations in which you experienced work as meaningful?** - Why did this work feel meaningful? What caused this work to feel meaningful? |
| 2.2 Outcomes of meaningful work | - **What was/were the outcome(s) of that meaningful work on yourself, your family, your patients and your practice?** |
| 3. Meaningless work | |
| 3.1 Meaningless work | - **Thinking about the last week or month of work, please tell me about one or more situations in which you experienced work as meaningless?** - Why did this work feel meaningless? What caused this work to feel meaningless? |
| 3.2. Outcomes of meaningless work | - **Think about yourself, your family, your patients, and your practice – who suffers when your work feels meaningless, and in what ways?** |
| 3.3 Proportion of meaningful vs. meaningless work | - IF NOT ALREADY MENTIONED, SPECIFICALLY ASK: **In your opinion, what proportion of your working time is spent on meaningful versus meaningless work?** |
| 4. Coping | |
| 4.1 Dealing with meaningless work | - We’ve talked about when and why you feel your work is meaningful or meaningless**. Are there things you do in your day-to-day work or even outside of your work to cope with meaningless work or to make your role as a GP/pediatrician feel more meaningful to you? If so, could you please tell me what you do?** - Do you talk to someone about the meaninglessness of your work? If so, with whom? And what do they say then? - How do your work colleagues feel about the issues we’ve been discussing in this interview? Is there any informal discussion about these issues at work? If so, what do people say? - Do you engage in primary-care-related activities outside of your paid work? For example, are you involved in professional groups or community initiatives related to health? If so, where and to what extent does this commitment contribute to the meaningfulness of their work? - IF NOT ALREADY MENTIONED, SPECIFICALLY ASK: **How effective are your strategies for dealing with meaningless work? How do these coping strategies impact you, your family, your patients, and your organization?** |
| 5. Healthcare system | |
| 5.1 Influence of the health system | - IF NOT ALREADY MENTIONED, SPECIFICALLY ASK: **How does the German healthcare system (e.g., the financing model) influence your experience with meaningful and meaningless work?** - In your opinion, what would have to change in the structure and organization of primary care in order to enable the doctors involved to feel meaning in their work? - **How could other professional groups (e.g., nurses) involved in the care of your patients support you in your day-to-day work (inside and outside your practice)?** - **In your opinion, what would have to change in the structure and organization of outpatient patient care?** |
| 6. Conclusion | |
| 6.1 Conclusion | - I realize that we can only cover so much in 60 minutes, so do you agree to being asked about a follow-up interview to share information we did not cover today? (Please note that agreeing to being asked about a follow-up interview is not the same as agreeing to such an interview; you can still decline the follow-up interview when asked about it) - Recommendation of other participants: Are there other people you would suggest for this interview? (Snowball method) - Would you like to read through the transcript of your interview before it is analyzed? - Would you like to receive a summary of the research results? |
